# Adjunctive Therapy With a *Perna canaliculus* and *Euphausia superba* Extract (EAB‐277^TM^) for Clinical Signs of Tracheal Collapse in Dogs: A Prospective, Randomized and Placebo‐Controlled Exploratory Trial

**DOI:** 10.1002/vms3.71159

**Published:** 2026-08-14

**Authors:** Wasutorn Yangwanitset, Somkiat Huaijantug, Mookmanee Taechikantaphat, Melanie Hezzell, Walasinee Sakcamduang

**Affiliations:** ^1^ Faculty of Veterinary Science Mahidol University Nakhon Pathom Thailand; ^2^ Department of Clinical Sciences and Public Health, Faculty of Veterinary Science Mahidol University Nakhon Pathom Thailand; ^3^ Bristol Veterinary School University of Bristol Bristol UK

**Keywords:** airway collapse, cough, exercise tolerance, krill oil, omega‐3, *Perna canaliculus*

## Abstract

**Background:**

Tracheal collapse is a common canine respiratory disease characterized by dorsoventral cartilage flattening, airway lumen narrowing and subsequent mucosal inflammation.

**Objectives:**

To investigate the efficacy of EAB‐277, a marine lipid extract combining *Perna canaliculus* (30 mg) and *Euphausia superba* (20 mg) as an adjunctive therapy in the management of tracheal collapse in dogs.

**Methods:**

Forty‐one dogs with confirmed tracheal collapse were randomly assigned to receive standard conventional medications plus either EAB‐277 (*n* = 21) or a placebo (*n* = 20). The EAB‐277 treatment protocol included a loading dose for the initial 14 days. Clinical signs and quality of life were assessed together with fluoroscopy, radiography, haematology and serum biochemistry on Days 0, 14, 28 and 56.

**Results:**

Dogs receiving EAB‐277 showed a significant improvement in exercise tolerance by Day 14. Concurrently, fluoroscopic analysis demonstrated significant reductions in the percentage change in tracheal luminal diameter across the cervical, thoracic inlet and intrathoracic regions by Day 14. Both groups showed significant improvements in cough scores throughout the study, with no statistical difference between the groups.

**Conclusions:**

Although conventional medical therapy effectively manages the clinical signs of tracheal collapse, particularly cough, this study demonstrates that EAB‐277 provides additional benefits by significantly improving exercise tolerance and reducing dynamic tracheal collapse at the cervical, thoracic inlet and intra‐thoracic regions, which might have the potential to enhance airway health.

## Graphical abstract

**Key findings**: Adjunctive EAB‐277^TM^ therapy may enhance airway health with the greatest improvements observed on Day 14 by improving exercise tolerance and reducing dynamic tracheal collapse.

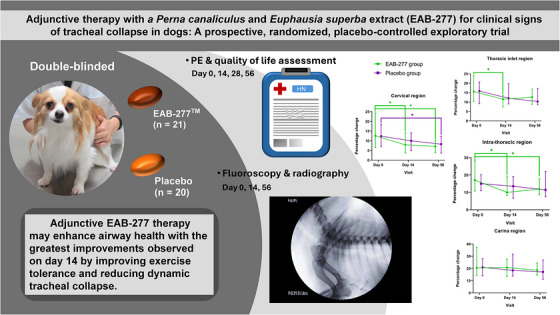

## 1 Introduction

Tracheal collapse is a progressive condition commonly found in middle‐aged dogs of small breeds. The exact cause of tracheal collapse is not well understood and is believed to involve multiple factors (Tappin 2016). Dorsoventral flattening of the trachea obstructs the flow of air through the respiratory tract, leading to chronic inflammation due to repetitive mucosal contact between the tracheal walls (Clarke 2018). Typical clinical signs of tracheal collapse include a persistent dry, paroxysmal ‘goose‐honk’ cough, mild to severe panting, exercise intolerance, cyanosis, syncope and respiratory distress (Della Maggiore 2020). Certain factors, such as heat, excitement, stress, inhalation of irritant and allergens or concurrent diseases, can exacerbate these clinical signs (Maggiore 2014; Beal 2013).

Several imaging techniques are employed for the diagnosis of tracheal collapse. Standard thoracic radiography is the most common tool used to investigate suspected cases of tracheal collapse. Typically, at least two‐view (orthogonal) radiographs, including inspiratory and expiratory lateral views, are obtained. However, the accuracy of diagnosis based solely on radiography ranges from 60% to 90%. Fluoroscopy is a superior imaging tool compared to radiography for detecting dynamic tracheal collapse, especially in the region of the carina (Tappin 2016; Macready et al. 2007; Yangwanitset et al. 2023). Although bronchoscopy is the human gold standard, its diagnostic utility in dogs relies on direct visual inspection rather than formal validation. Despite providing unparalleled mucosal detail, the necessity of general anaesthesia may paradoxically mask dynamic collapse by altering respiratory pressure gradients (Heyer et al. 2007; Johnson et al. 2015).

Antinol EAB‐277 is a natural supplement with anti‐inflammatory properties. It contains 30 mg of lipid fractions sourced from *Perna canaliculus* (green‐lipped mussel) and 20 mg of high phospholipid from *Euphausia superba* (krill). These lipid fractions are rich in long‐chain *ω*‐3 polyunsaturated fatty acids (PUFAs), known for their anti‐inflammatory effects. The oil extract from New Zealand green‐lipped mussels has been used to manage inflammatory conditions in dogs, especially canine osteoarthritis (OA) (Vijarnsorn et al. 2019), whereas krill oil has demonstrated the ability to suppress inflammatory cytokines in a mouse model of arthritis (Ierna et al. 2010). Brachycephalic obstructive airway syndrome in dogs displays features similar to those seen in obstructive sleep apnea syndrome (OSAS) in humans, leading to an elevation in oxidative stress caused by hypoxia (Hendricks 1992; Hendricks et al. 1987; Eisele et al. 2015; Lavie 2015). Tracheal collapse may occur as a secondary change associated with BOAS, possibly resulting from chronically elevated negative intrathoracic pressure (Krainer and Dupré 2022). A study conducted on dogs with tracheal collapse revealed that EAB‐277 has the potential to alleviate oxidative stress and reduce inflammatory markers (Mektrirat et al. 2022).

Management of tracheal collapse is primarily palliative, focusing on alleviating clinical signs through environmental controls and antitussive medications. Although other therapeutics, such as glucocorticoids or bronchodilators, may be used, their role is typically for management of specific concurrent inflammatory airway diseases rather than the structural collapse itself (Carr et al. 2022). However, there is limited information available on the effectiveness of adjunctive nutraceuticals like EAB‐277. Therefore, the objective of this study was not to test EAB‐277 as a primary treatment for the structural component of tracheal collapse. Instead, this study was designed as an exploratory trial to determine if EAB‐277, when used as an adjunct to conventional medication, provided any measurable additional benefit. We hypothesized that administration of EAB‐277 as adjunctive supplement might result in significant improvements in clinical signs (primarily cough and exercise intolerance) and a reduced degree of tracheal collapse on diagnostic imaging.

## 2 Materials and Methods

### 2.1 Dogs

The single‐centre, prospective, randomized parallel study was approved by the Faculty of Veterinary Science, Mahidol University‐Institute Animal Care and Use Committee, Thailand (COA No. MUVS‐2020‐01‐02). Written informed consent was obtained from the owners for the participation of their animals in this study. This study was conducted at Prasu Arthorn Veterinary Teaching Hospital, Faculty of Veterinary Science, Mahidol University, Thailand. All dogs were randomly allocated into two groups as follows: EAB‐277 and placebo using block randomization. The inclusion criteria for this study were client‐owned dogs of any sex, age or breed with a confirmed diagnosis of tracheal collapse through either radiography or fluoroscopy. Dogs with myxomatous mitral valve disease (MMVD) Stage B1 or lower, diagnosed according to the American College of Veterinary Internal Medicine (ACVIM) consensus statement (Keene et al. 2019), were eligible for the study. Prior to participating in the study, informed consent was obtained from the owners. Dogs were excluded from the study if tracheal collapse could not be detected by fluoroscopy conducted by the investigators or if they had had congestive heart failure, MMVD stage B2 or higher (determined by cardiomegaly using three criteria: vertebra heart size (VHS) greater than 10.5 (Hansson et al. 2005), left atrial‐to‐aortic root ratio (LA/Ao) ≥1.6, (Hansson et al. 2002) and body weight normalized left ventricular internal diameter in diastole (LVIDDN) ≥1.7 (Cornell et al. 2004), congenital or other acquired heart diseases, heartworm disease or systemic conditions affecting the respiratory tract. All dogs received conventional medications for tracheal collapse, including antitussive drugs, bronchodilators and mucolytic drugs, in addition to either the marine lipid extract EAB‐277 or a placebo for 56 days. The dosage regimen consisted of two capsules (each capsule contains 30 mg of *P. canaliculus* and 20 mg of *E. superba*) per day as a loading dose for the first 14 days, followed by one capsule per day as a maintenance dose until Day 56. Throughout the study period, owners participated in history‐taking sessions, whereas the dogs underwent physical examination, and blood collection (5 mL) for haematology (complete blood count) and serum biochemistry (blood urea nitrogen [BUN], creatinine, alanine aminotransferase, alkaline phosphatase, total protein and albumin) on Days 0 (visit 1), 14 (visit 2), 28 (visit 3) and 56 (visit 4), respectively. These blood tests were performed for screening purposes, to assess overall health and monitor for any changes throughout the study period.

### 2.2 Questionnaires

On Day 0 (baseline), the clinical status of the dogs was assessed using a questionnaire administered to the owners of the dogs (Table S1). The questionnaire utilized an ordinal scale on the basis of Häggström et al. (2008) protocol to score clinical signs that impact the dog's quality of life. Changes in these clinical signs were considered the primary outcome of the study. To track changes over time, the same questionnaire was completed by the owners on Days 14, 28 and 56 to determine if clinical signs had improved, remained the same or worsened compared to the previous visit (Table S2).

### 2.3 Imaging Study

Non‐sedated dogs were positioned for imaging using a Philips Medical Systems Bucky Diagnost CS (Hamburg, Germany) radiographic system. Right lateral radiographs were obtained during both inspiratory and expiratory phases, and dorsoventral radiographs were obtained during maximum inspiration, whenever possible. Thoracic radiography was performed to screen for concurrent cardiopulmonary diseases and to evaluate cardiac dimensions. The tracheal diameter changes from radiography were recorded. Fluoroscopy was conducted with the dogs positioned in right lateral recumbency, observing tidal respiration and inducing a cough through tracheal manipulation to confirm tracheal collapse. At least three respiratory cycles were recorded during the procedure. Fluoroscopy using the Philips BV Libra system (Hamburg, Germany) captured images during full inspiration and expiration phases. The classification of tracheal collapse was based on the criteria established by Tanger and Hobson (1982) in 1982, with grades I–IV representing reductions in luminal diameter of <25%, 25% to <50%, 50% to <75% and 75%–90%, respectively. For Grade I collapse, the percentage change in tracheal luminal diameter had to exceed 16.4%, based on the maximum percentage fluctuation observed in the tracheal diameter of healthy dogs (Scherf et al. 2020). The trachea was divided into four regions: cervical, thoracic inlet, intra‐thoracic and carina, using the same reference vertebral range as a previous study (Yangwanitset et al. 2023). The tracheal height was measured at maximum inspiration and maximum expiration using ImageJ software. The percentage difference between these measurements was calculated and averaged over three repetitions. All measurements were performed by a single observer (W.Y.), who was blinded to the dogs’ identity to minimize bias. The calculation was performed using the formula % tracheal height = [(mean tracheal height at maximum inspiration − mean tracheal height at max expiration)/mean tracheal height at maximum inspiration] × 100 (Scherf et al. 2020; Leonard et al. 2009). Radiography and fluoroscopy were performed on Days 0, 14 and 56. Echocardiographic examinations were conducted without sedation on Day 0 using a GE Vivid E9 (Horten, Norway). The examinations included 2D and Doppler echocardiography to assess any abnormalities in the heart. The shape and size of the heart were evaluated using 2D ultrasonography in various views, such as the right parasternal long axis 4‐chamber view, right parasternal long axis 5‐chamber view and right parasternal short axis views at the levels of the papillary muscles, chordae tendineae, heart base and aorta and pulmonary artery. These evaluations were performed with the dog in a right lateral recumbent position.

### 2.4 Statistical Analysis

The sample size was calculated using G*Power version 3.1.9.4 (Franz Faul, Universität Kiel, Germany), assuming a large effect size (Cohen's *d* = 1.0), with a significance level (*α*) of 0.05 and statistical power (1 − *β*) of 0.8, a minimum of 17 dogs per group was required to detect a significant difference. Computerized statistical software (SPSS 28.0 for Windows, Chicago, IL, USA) was used for analyses. The normality of variables obtained from each group on each examination day (imaging results and blood test results) was assessed using the Shapiro–Wilk test. Normally distributed data were presented as mean ± standard deviation (SD), whereas non‐normally distributed data and ordinal data were presented as median with interquartile ranges. To analyse changes in quality of life between visits, Wilcoxon signed‐rank tests were performed. Independent *t*‐tests or Mann–Whitney‐*U* tests were used to compare variables between groups at each examination. For comparisons within groups across visits, repeated measured ANOVAs or Friedman tests were employed, depending on the appropriateness of the data. A significance level of *p* < 0.05 was used to determine statistical significance.

## 3 Results

### 3.1 Demographic Characteristics

Between August 2020 and March 2022, a total of 53 dogs diagnosed with tracheal collapse were initially enrolled in this study. However, 12 dogs were subsequently excluded from the study for various reasons, including the absence of dynamic changes in tracheal diameter (*n* = 9), non‐compliance (*n* = 1), loss to follow‐up (*n* = 1) and diarrhoea believed to be caused by dietary changes during the study (*n* = 1). Therefore, the final analysis included a total of 41 dogs that successfully completed the study. Among the included dogs, there were 22 males and 19 females (13 neutered males and 12 spayed females). The distribution of breeds was as follows: 18 Pomeranians (43.9%), 10 Pugs (24.4%), 5 Chihuahuas (12.0%), 4 Shi‐Tzu (9.8%), 2 Yorkshire Terriers (4.9%) and 1 (2.4%) each of French bulldog and English mastiff. The mean ± SD age was 7.9 ± 3.7 years, and their average weight was 6.5 ± 3.2 kg. The median body condition score (BCS) (9‐grade system) was 7/9 (5,8). Six dogs had MMVD stage B1 (EAB‐277 group, *n* = 4; placebo group, *n* = 2). No significant differences in baseline demographic characteristics were observed between the two groups (Table 1). The medication history of both groups can be found in Tables S3 and S4. After the completion of the clinical trial, three dogs (EAB group *n* = 2, placebo group *n* = 1) underwent surgical procedures based on clinical findings to further investigate and correct the underlying disease.

**TABLE 1:** Demographic and baseline data of the study participants separated for each group.

	EAB‐277	Placebo	*p* value
Dog characteristics			
Number	21 (MMVD B1 *n* = 4)	20 (MMVD B1 *n* = 2)	0.34
Sex (F/M)	10/11	12/8	0.65
Gonadectomy (N/Y)	6/15	10/10	0.21
Age (years)	8.4 ± 3.8	7.3 ± 3.5	0.35
Body weight (kg)	7.4 ± 3.7	5.6 ± 2.2	0.08
Body condition score (9 Grade)	7 (5,8)	6 (5,8)	0.33
Breed	9PP, 6PU, 2CH, 4OT	9PP, 4PU, 3CH, 4OT	
Quality of life variables			
Exercise intolerance (very good/good/moderate) (%)	3/15/3 (14.3/71.4/14.3)	7/9/4 (35.0/45.0/20.0)	0.301
Demeanor (very good/good/moderate) (%)	19/1/1 (90.5/4.8/4.8)	15/4/1 (75.0/20.0/5.0)	0.252
Appetite (very good/good/moderate/poor) (%)	3/16/1/1 (14.3/76.2/4.8/4.8)	8/9/2/1 (40.0/45.0/10.0/5.0)	0.252
Respiratory effort (very good/good/moderate) (%)	8/12/1 (38.1/57.1/4.8)	7/11/2 (35.0/55.0/10.0)	0.569
Cough (very good/good/moderate/poor) (%)	5/6/6/4 (23.8/28.6/28.6/19.0)	3/2/10/5 (15.0/10.0/50.0/25.0)	0.113
Nocturnal dyspnea (good/moderate/poor)(%)	14/5/2 (66.7/23.8/9.5)	11/5/4 (55.0/25.0/20.0)	0.238

*Note*: Results were presented as mean ± standard deviation (SD) for normally distributed variables and median with interquartile ranges for non‐normally distributed variables. The quality of life variables between the two groups were compared using the Mann–Whitney *U* test.

Abbreviation: MMVD, myxomatous mitral valve disease; PP, Pomeranians; PU, Pugs; CH, Chihuahuas; OT, other breeds.

### 3.2 Questionnaire Results

At baseline (Day 0), there were no significant differences in the quality of life results between the two groups (Table 1). When comparing the variables between the two groups on Day 14 relative to baseline, based on owner reports, no significant differences were found in any of the variables. However, within‐group comparisons revealed that the EAB‐277 group showed a significant improvement in exercise tolerance (*p* = 0.008), whereas both the EAB‐277 and placebo groups demonstrated a marked improvement in cough (*p* = 0.005 and 0.001, respectively; Table 2A).

**TABLE 2:** Quality of life comparisons within‐group and between‐group between baseline data and Day 14.

Variables	Group	*N*	Deteriorated	Unchanged	Improved	Within‐group comparison	Between‐group comparison
**A. Day 14 to baseline**							
Exercise intolerance	EAB‐277	21	0	14	7	0.008	0.301
	Placebo	20	4	9	7	0.285	
Demeanor	EAB‐277	21	4	15	2	0.589	0.252
	Placebo	20	1	17	2	0.564	
Appetite	EAB‐277	21	3	12	6	0.248	0.252
	Placebo	20	5	14	1	0.102	
Respiratory effort	EAB‐277	21	4	12	5	0.705	0.569
	Placebo	20	2	12	6	0.157	
Cough	EAB‐277	21	2	6	13	0.031	0.113
	Placebo	20	0	10	10	0.001	
Nocturnal dyspnea	EAB‐277	21	4	11	6	0.739	0.739
	Placebo	20	1	13	6	0.058	
B. **Day 28 to baseline**							
Exercise intolerance	EAB‐277	21	3	11	7	0.166	1.000
	Placebo	20	4	11	5	0.951	
Demeanor	EAB‐277	21	3	16	2	1.000	0.637
	Placebo	20	1	17	2	0.564	
Appetite	EAB‐277	21	2	15	4	0.414	0.058
	Placebo	20	4	9	7	0.285	
Respiratory effort	EAB‐277	21	5	8	8	0.593	0.457
	Placebo	20	3	11	6	0.480	
Cough	EAB‐277	21	5	8	8	0.028	0.354
	Placebo	20	3	11	6	0.002	
Nocturnal dyspnea	EAB‐277	21	2	14	5	0.414	0.356
	Placebo	20	2	11	7	0.083	
C. **Day 56 to baseline**							
Exercise intolerance	EAB‐277	20	2	11	7	0.083	0.926
	Placebo	20	5	7	8	0.405	
Demeanor	EAB‐277	20	4	14	2	0.739	0.496
	Placebo	20	2	14		0.414	
Appetite	EAB‐277	20	2	11	7	0.132	0.507
	Placebo	20	5	10	5	0.957	
Respiratory effort	EAB‐277	20	2	11	7	0.059	0.105
	Placebo	20	2	14	4	0.414	
Cough	EAB‐277	20	2	11	7	0.078	0.878
	Placebo	20	2	14	4	0.004	
Nocturnal dyspnea	EAB‐277	20	1	12	7	0.059	0.245
	Placebo	20	2	11	7	0.057	

*Note*: The data were reported in three categories (deteriorated, unchanged, and improved). Comparisons between the two groups were tested using Mann–Whitney *U* test. Comparisons between visits within subjects in each group were conducted using Wilcoxon signed‐rank test.

On Day 28, both the EAB‐277 and placebo groups exhibited significant improvements in the cough variable compared to baseline (*p *= 0.028 and 0.002, respectively). However, no significant differences were observed in other variables in both within‐group and between‐group comparisons (Table 2B).

On Day 56, the placebo group showed a significant improvement in the cough variable compared to baseline (*p* = 0.004), whereas the EAB‐277 group did not (*p* = 0.078). No significant differences were observed in the other variables in both within‐group and between‐group comparisons (Table 2C).

### 3.3 Imaging Results

Fluoroscopy served as the primary imaging modality for evaluating tracheal collapse due to its superior ability to capture dynamic changes in tracheal diameter during respiration. Detailed radiographic findings are provided in Tables S5–S11. The distribution of dogs based on the severity of tracheal collapse, as determined by fluoroscopy, is presented in Table 3. Table 4 further describes the number and grading of collapsing tracheal areas observed through fluoroscopy.

**TABLE 3:** Number of dogs categorized by collapsing areas.

Number of collapsing areas	EAB‐277	Placebo
Focal collapse (1 area)	6	7
Multifocal (2 areas)	7	5
Multifocal (3 areas)	3	5
Generalized collapse (4 areas)	5	3

**TABLE 4:** Number of dogs categorized by grading of collapsing areas.

	EAB‐277					Placebo					
Regions/Grade	I	II	III	IV	All	I	II	III	IV	all	*p* value
Cervical	7	3	—	—	10	5	2	—	—	7	0.949
Thoracic inlet	6	3	—	—	9	4	4	1	—	9	0.462
Intra‐thoracic	—	7	—	—	7	4	4	1	—	9	0.059
Carina	7	8	2	—	17	8	4	1	—	13	0.543

At baseline (Day 0), there were no significant differences in the percentage change of tracheal lumen between the EAB‐277 and placebo groups in any of the tracheal areas (cervical, thoracic inlet, intra‐thoracic or carina) (Table 5). Comparisons of tracheal height percentage difference obtained by fluoroscopy between full inspiration and expiration phases, divided into 4 regions for both groups, can be found in Table S12, where no significant findings were found between groups at any of the regions or between groups at any of the visits.

**TABLE 5:** Percentage changes obtained from fluoroscopy were reported separately in each group.

	EAB‐277			Placebo		
	Day 0	Day 14	Day 56	Day 0	Day 14	Day 56
Cervical	12.58 (6.54,24.40)^a,b^	7.88 (3.66,14.21)^a^	7.17 (3.85,10.40)^b^	12.39 (7.15,17.57)^c^	9.98 (4.98,14.14)	8.27 (3.65,12.23)^c^
Thoracic inlet	15.59 (10.35,24.03)^d^	11.52 (7.37,13.49)^d^	12.61 (8.79,16.95)	15.87 (9.15,20.67)	12.03 (9.03,19.78)	10.29 (8.54,17.26)
Intra‐thoracic	17.09 (10.80,28.25)^e,f^	10.02 (8.23,19.01)^e^	11.89 (8.68,17.87)^f^	15.00 (10.97,20.15)	13.57 (6.67,19.09)	11.44 (7.58,22.04)
Carina	20.47 (14.31,37.43)	20.81 (13.49,27.70)	18.50 (14.60,24.50)	20.99 (14.01,28.05)	18.43 (13.93,31.83)	17.23 (11.18,26.99)

*Note*: Comparisons between visits within subjects in each group were conducted using Wilcoxon signed‐rank test. The values with same superscript letters in a column of each group are significantly different (*p* < 0.05).

**FIGURE 1:**
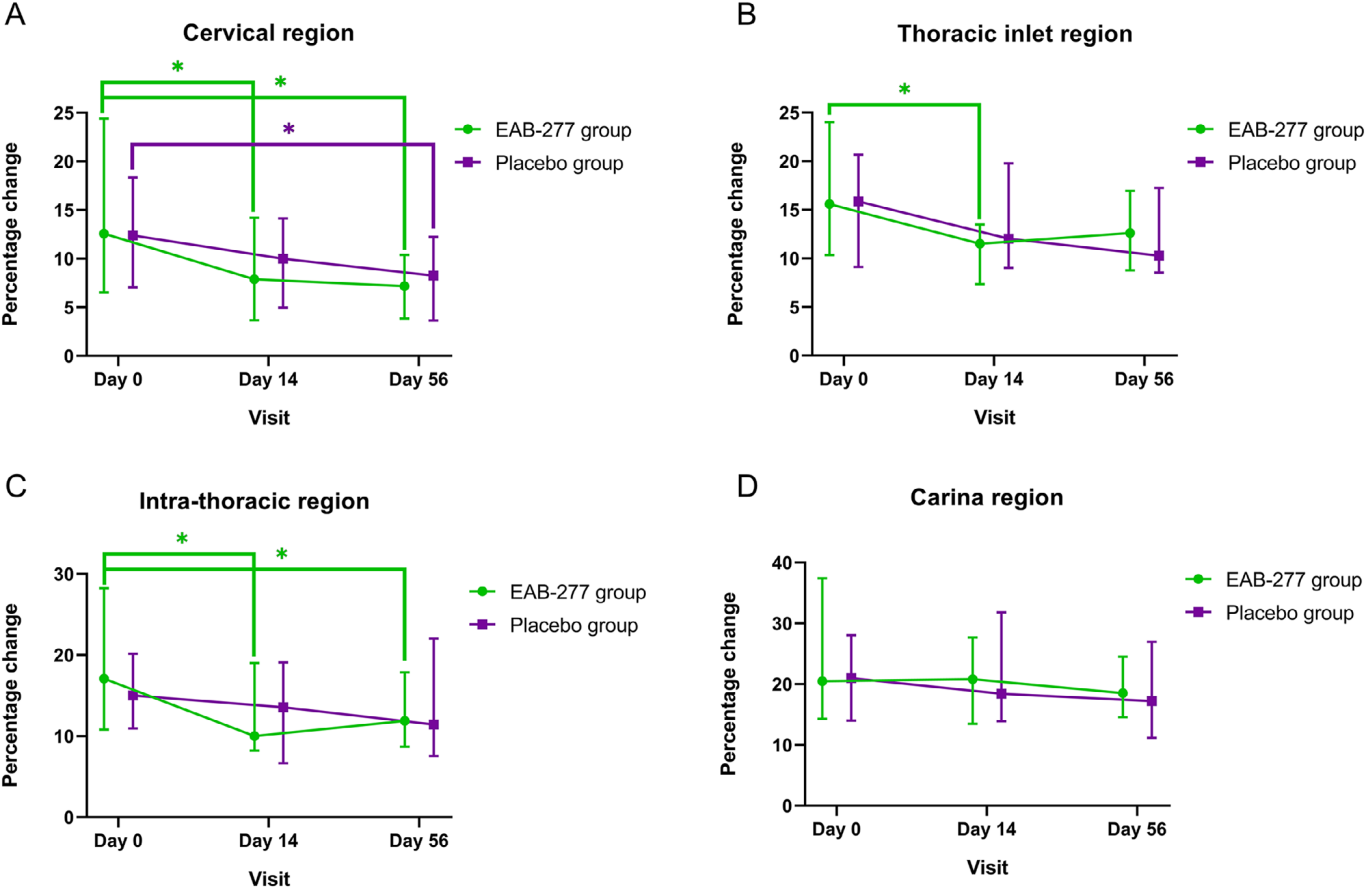
The line graphs show the median and interquartile (Q1 and Q3) of the tracheal height reduction percentages obtained by fluoroscopy between maximum inspiration and expiration phases were plotted for each region, including (A) cervical region, (B) thoracic inlet region, (C) intra‐thoracic region and (D) carina region. Asterisk indicates a significant difference (*p* < 0.05).

Within the EAB‐277 group, the percentage change of tracheal lumen decreased significantly from baseline to Day 14 in the cervical region (*p* = 0.016, Figure 1A), thoracic inlet region (*p* = 0.003, Figure 1B) and the intra‐thoracic region (*p* = 0.009, Figure 1C). Significant decreases were also observed between Days 0 and 56 in the cervical region (*p* = 0.019, Figure 1A) and intra‐thoracic region (*p* = 0.008, Figure 1C). No significant difference was observed in the carina region at any time point (Figure 1D).

In the placebo group, a significant decrease in the percentage change of tracheal lumen was observed from baseline to Day 56 in the cervical region (*p* = 0.017, Figure 1A).

Representative fluoroscopic images obtained during both inspiration and expiration at Days 0 and 14 are provided as Figure S1 to illustrate the changes in tracheal lumen observed following treatment.

### 3.4 Blood Profiles

BUN concentrations were significantly higher in the EAB‐277 group (median 23 mg/dL, interquartile [IQR] 18–29) compared to the placebo group (median 17 mg/dL, IQR 13–20; *p* = 0.034). However, this statistical difference lacked clinical significance, as all individual haematological and biochemical values for every dog remained strictly within established reference intervals. No other significant differences were detected across the remaining laboratory variables (Table S13).

## 4 Discussion

This prospective exploratory trial was designed to investigate the adjunctive benefits of the anti‐inflammatory marine lipid extract EAB‐277 in dogs with tracheal collapse already receiving conventional medical management. The study failed to demonstrate a significant superiority of EAB‐277 over placebo for the primary outcome measure, owner‐assessed cough scores, at any follow‐up visit.

A significant improvement in cough was observed in both groups, suggesting a strong therapeutic effect from the concurrent conventional medications and a potential placebo effect. This potent confounding factor makes it challenging to isolate any adjunctive benefits of EAB‐277. Although our hypothesis focused on EAB‐277's role in mitigating the secondary tracheitis resulting from mucosal friction, the intensive cough management in both groups may have masked any specific anti‐inflammatory contribution.

The demographic profile of the study population primarily middle‐aged, obese dogs; aligns with established risk factors for both tracheal collapse and comorbid OA (Macready et al. 2007; Vijarnsorn et al. 2019; Johnson and Pollard 2010; Taylor‐Brown et al. 2015; Sanderson 2012; Clements et al. 2006). A key finding was the significant ‘within‐group’ improvement in exercise tolerance exclusively in the EAB‐277 group at Day 14, coinciding with the loading dose period. Given the anti‐inflammatory properties of omega‐3 fatty acids, this improvement likely reflects a dual‐action benefit: a reduction in tracheal luminal inflammation and a systemic analgesic effect on subclinical OA pain. Although exercise intolerance in this demographic is multifactorial, the tangible quality‐of‐life improvement achieved by Day 14 underscores the systemic efficacy of EAB‐277. Consequently, future investigations should transition from a localized respiratory focus to a broader evaluation of its impact on the systemic inflammatory milieu in geriatric, overweight patients (Vijarnsorn et al. 2019). Regarding the fluoroscopic findings, we must address the concern that measuring luminal diameter is irrelevant for a structural disease. We respectfully posit that these measurements remain relevant under our hypothesis of secondary inflammation. EAB‐277 is not expected to reverse the underlying chondromalacia; rather, we propose that by reducing mucosal inflammation and oedema, airway function may be preserved. The significant ‘within‐group’ reduction in tracheal lumen percentage change in the EAB‐277 group at Day 14 in three regions supports this potential mechanism. These findings suggest a potential physiological improvement in airway function resulting from reduced inflammation, rather than reversal of structural abnormalities.

Fluoroscopic imaging in this study confirmed that the carina was the most severely collapsed and most commonly affected area of the trachea, consistent with previous findings (Macready et al. 2007; Kim et al. 2024). Notably, neither group exhibited significant dynamic change in tracheal lumen diameter in this region throughout the study period. In a previous study, the tracheal diameter/thoracic inlet distance ratio (TD/TI ratio) measured from a single radiographic image that did not change significantly over the 5‐week study period between the EAB‐277 and placebo groups (Mektrirat et al. 2022). The present study, in contrast, leveraged fluoroscopy's ability to capture dynamic changes in tracheal diameter during different respiratory phases, providing a more comprehensive assessment of this condition. This dynamic assessment allowed for a more precise evaluation of tracheal collapse and its response to treatment with EAB‐277.

The observed reduction in cough scores across both cohorts initially suggests that the standardized baseline therapy comprising antitussives and bronchodilators was the primary driver of clinical stabilization. However, the divergence at Day 56, where only the placebo group maintained statistical significance, warrants critical interrogation. This disparity likely represents a Type II error rather than a lack of efficacy; it is biologically improbable that the addition of EAB‐277 would antagonize the benefits of standard care (Mektrirat et al. 2022). A more plausible explanation lies in the transition from the loading dose to the maintenance dose. The diminution of significant effects in the treatment group post‐Day 14 suggests that although the initial high‐dose saturation provided potent anti‐inflammatory support, the subsequent maintenance dose may have fallen below the therapeutic threshold required to suppress chronic airway irritation in these specific subjects. Furthermore, the inherent variability of cough frequency in dynamic tracheal collapse may have masked a sustained clinical benefit, emphasizing that although standard therapy manages acute symptoms, the long‐term immunomodulatory role of EAB‐277 requires further dose‐escalation studies to be fully elucidated.

The observed improvements in cough‐related quality of life scores appeared temporally independent of structural changes in tracheal lumen patency. This dissociation aligns with previous findings where no direct correlation was stablished between cough severity and the anatomical degree of collapse as assessed via fluoroscopy (Kim et al. 2024). Two hypotheses may elucidate the observed reduction in luminal percentage change. Although this may represent stochastic variation inherent to dynamic airway imaging, an alternative biological mechanism warrants consideration (Torrego et al. 2006; Grillo et al. 2022). The potential for EAB‐277 to mitigate tracheal mucosal inflammation and oxidative stress may contribute to structural stabilization or improved mucosal integrity (Mektrirat et al. 2022; Calder 2015). However, the lack of a superior reduction in cough frequency relative to the placebo group suggests that structural lumen improvements do not immediately attenuate the hypersensitized cough reflex typical of chronic collapse. This confirms that although EAB‐277 may support airway architecture, the clinical manifestation of ‘cough’ remains a complex, multifactorial phenomenon that persists beyond immediate anatomical shifts.

The treatment approach for canine tracheal collapse aims to manage clinical signs, enhance quality of life and reduce the severity of tracheal collapse. This study demonstrated short‐term improvement, particularly on Day 14, in the EAB‐277 group, possibly due to the loading dose. However, the long‐term results on Day 56 did not show a prolonged effect. Therefore, further investigation is warranted to explore the potential benefits of continuing the supplement at the loading dose (2 capsules/day) after Day 14 in affected dogs. Another possible explanation for lack of long‐term effectiveness of EAB‐277 could be the success reduction of aggravating factors in both groups during the study, leading to the absence of inflammation after 2 weeks of treatment.

The findings of this study must be interpreted with significant caution due to several major limitations. First, this trial enrolled dogs without bronchoscopic evaluation to rule out concurrent lower airway diseases (e.g., chronic bronchitis and bronchomalacia). These conditions can independently cause coughing and confound the assessment. Second, the inclusion of brachycephalic breeds (Pugs) known for brachycephalic airway syndrome introduces confounding upper airway factors that affect clinical signs. Moreover, after the completion of the clinical trial, three Pugs underwent endoscopic investigation for laryngeal saccule observation and surgical correction procedures for conditions, such as alarplasty and staphylectomy. These concurrent diseases represent upper respiratory tract disorders that can significantly affect clinical signs, which is also a limitation of the study as it can impact the questionnaire results, especially in relation to cough variables. However, even after excluding the three subjects that underwent surgery or all Pugs, the EAB‐277 group maintained the same trend, demonstrating improved exercise intolerance and a significant reduction in the percentage change in tracheal lumen (Tables S14–S15). Third, our primary outcome measures relied on subjective owner‐reported quality of life questionnaires, which are prone to placebo effects, and non‐sedated imaging, which introduces measurement variability. In contrast, procedures, such as computerized tomography or endoscopy, can be performed on anesthetized animals, although anaesthesia carries its own risks (Johnson et al. 2015; Williams et al. 2016). In addition, other objective tests, such as pulmonary function testing for analysis of tidal breathing flow‐volume loops, could be beneficial for evaluating the degree and progression of the disease. These non‐invasive methods do not require restraint during the procedure, unlike imaging studies (Amis and Kurpershoek 1986; Rozanski and Hoffman 1999; McKiernan and Johnson 1992). Finally, the high dropout rate (12/53) also reduced the final sample size and statistical power.

In conclusion, this study provides evidence for the potential benefits of EAB‐277 as an adjunctive therapy for tracheal collapse in dogs. Notably, within the first 2 weeks of treatment, during which a loading dose of EAB‐277 was administered, significant improvements in exercise tolerance and airway health were observed. Fluoroscopic evaluation revealed that EAB‐277 may lead to more rapid and targeted improvements in tracheal collapse compared to standard treatment alone. Although further research is needed to fully elucidate the long‐term effects and optimal dosing strategies of EAB‐277, this study highlights its potential as a valuable tool in managing this challenging condition and improving the quality of life for affected dogs.

## Author Contributions

**Wasutorn Yangwanitset**, **Somkiat Huaijantug**, **Mookmanee Taechikantaphat**, and **Walasinee Sakcamduang** conceptualized the project. Walasinee Sakcamduang acquired funding and supervised the project. Wasutorn Yangwanitset, Somkiat Huaijantug, Mookmanee Taechikantaphat, and Walasinee Sakcamduang managed the coordination and execution of activities, including the formal analysis. Somkiat Huaijantug, Mookmanee Taechikantaphat, and Walasinee Sakcamduang provided resources. Wasutorn Yangwanitset wrote original draft, which underwent review, editing, and conclusion by Somkiat Huaijantug, Mookmanee Taechikantaphat, **Melanie Hezzell** and Walasinee Sakcamduang. All authors participated in the development of the manuscript, reviewed, and approved the final submission.

## Funding

This study was supported by Pharmalink International Limited, with an address at the third Floor, 31 C‐D Wyndham Street, Central, Hong Kong. The funder was not involved in the study design, collection, analysis, interpretation of data, the writing of this article or the decision to submit it for publication.

## Ethics Statement

All protocols in this study were approved by the Faculty of Veterinary Science, Mahidol University‐Institute Animal Care and Use Committee, Thailand (COA No. MUVS‐2020‐01‐02), in compliance with the Animals for Scientific Purposes Act, B.E. 2558 (A.D. 2015), Kingdom of Thailand. Written informed consent was obtained from the owners for the participation of their animals in this study.

All methods were performed in accordance with the relevant guidelines and regulations.

All methods are reported in accordance with ARRIVE guidelines (https://arriveguidelines.org) for the reporting of animal experiments.

## Conflicts of Interest

The authors declare no conflicts of interest.

## Supporting information




**Supporting File 01**: vms371159‐sup‐0001‐FigureS1.tif

**Supporting File 02**: vms371159‐sup‐0002‐Tables.docx

**Supporting File 03**: vms371159‐sup‐0003‐SupMat‐Figure‐Legends.docx

## Data Availability

The datasets generated for this study are available on request to the corresponding author.
