# Supplementary material for: Adjunctive Therapy With a Perna canaliculus and Euphausia superba Extract (EAB‐277TM) for Clinical Signs of Tracheal Collapse in Dogs: A Prospective, Randomized and Placebo‐Controlled Exploratory Trial
Source: Vet Med Sci. 2026 Aug 14;12(5):e71159. doi: 10.1002/vms3.71159 (PMC13475633; doi:10.1002/vms3.71159)
Supplement: Supplementary file 1 [file VMS3-12-e71159-s001.docx]

Supplementary Material

**Adjunctive therapy with a Perna canaliculus and Euphausia superba extract (EAB-277™) for clinical signs of tracheal collapse in dogs: A prospective, randomized, placebo-controlled exploratory trial**

# 1. Supplementary Tables

**Supplementary Table 1.** Questionnaire for evaluating quality of life on day 0

| Variable | Score | | Clinical Correlate |
| --- | --- | --- | --- |
| Exercise tolerance | □ | 1 (Very good) | Dog moved around with ease, was able to fully exercise |
|  | □ | 2 (Good) | Dog moved around with ease, was not able to fully exercise; ability to run was reduced |
|  | □ | 3 (Moderate) | Dog was less active than normal, moved around a few times per day, avoided long walks |
|  | □ | 4 (Poor) | Dog was inactive and would only get up to eat, drink, or urinate |
| Demeanor | □ | 1 (Very good) | Alert, responsive |
|  | □ | 2 (Good) | Mildly lethargic |
|  | □ | 3 (Moderate) | Moderately lethargic |
|  | □ | 4(Quite poor) | Minimally responsive |
|  | □ | 5 (Poor) | Unresponsive |
| Appetite | □ | 1 (Very good) | Increased |
|  | □ | 2 (Good) | Normal |
|  | □ | 3 (Moderate) | Decreased 1-30 % |
|  | □ | 4 (Poor) | Markedly decreased more than 30 % |
| Respiratory effort | □ | 1 (Very good) | Normal |
|  | □ | 2 (Good) | Mildly increased rate or effort |
|  | □ | 3 (Moderate) | Moderately labored |
|  | □ | 4 (Poor) | Severe respiratory distress |
| Coughing | □ | 1 (Very good) | None |
|  | □ | 2 (Good) | Occasional (a few times a week) |
|  | □ | 3 (Moderate) | Frequent (a few times a day) |
|  | □ | 4 (Poor) | Persistent (frequently during the day) |
| Nocturnal dyspnea/cough | □ | 1 (Good) | None |
|  | □ | 2 (Moderate) | Dog coughed from time to time during the night, but no other clinical signs of dyspnea or restlessness were present |
|  | □ | 3 (Poor) | Dog coughed consistently, increased respiratory effort or restlessness during the night |

Häggström’s et al (2008) protocol ^1^

**Supplementary Table 2.** Questionnaire for evaluating quality of life on day 14, 28 and 56

| Variable | Score | | Clinical Correlate | |
| --- | --- | --- | --- | --- |
| Exercise tolerance | □ | 1 (Very good) | Dog moved around with ease, was able to fully exercise | |
|  | □ | 2 (Good) | Dog moved around with ease, was not able to fully exercise; ability to run was reduced | |
|  | □ | 3 (Moderate) | Dog was less active than normal, moved around a few times per day, avoided long walks | |
|  | □ | 4 (Poor) | Dog was inactive and would only get up to eat, drink, or urinate | |
|  | When comparing with last visit, How the symptom change?  □ markedly improved □ improved □ not change □ Worse □ Worst | | | |
| Demeanor | □ | 1(Very good) | Alert, responsive | |
|  | □ | 2 (Good) | Mildly lethargic | |
|  | □ | 3 (Moderate) | Moderately lethargic | |
|  | □ | 4 (Quite poor) | Minimally responsive | |
|  | □ | 5 (Poor) | Unresponsive | |
|  | When comparing with last visit, How the symptom change?  □ markedly improved □ improved □ not change □ Worse □ Worst | | | |
| Appetite | □ | 1 (Very good) | Increased | |
|  | □ | 2 (Good) | Normal | |
|  | □ | 3 (Moderate) | Decreased 1-30 % | |
|  | □ | 4 (Poor) | Markedly decreased more than 30 % | |
|  | When comparing with last visit, How the symptom change?  □ markedly improved □ improved □ not change □ Worse □ Worst | | | |
| Respiratory effort | □ | 1 (Very good) | | Normal |
|  | □ | 2 (Good) | | Mildly increased rate or effort |
|  | □ | 3 (Moderate) | | Moderately labored |
|  | □ | 4 (Poor) | | Severe respiratory distress |
|  | When comparing with last visit, How the symptom change?  □ markedly improved □ improved □ not change □ Worse □ Worst | | | |
| Coughing | □ | 1 (Very good) | | None |
|  | □ | 2 (Good) | | Occasional (a few times a week) |
|  | □ | 3 (Moderate) | | Frequent (a few times a day) |
|  | □ | 4 (Poor) | | Persistent (frequently during the day) |
|  | When comparing with last visit, How the symptom change?  □ markedly improved □ improved □ not change □ Worse □ Worst | | | |
| Nocturnal dyspnea/cough | □ | 1 (Good) | | None |
|  | □ | 2 (Moderate) | | Dog coughed from time to time during the night, but no other clinical signs of dyspnea or restlessness were present |
|  | □ | 3 (Poor) | | Dog coughed consistently, increased respiratory effort or restlessness during the night |
|  | When comparing with last visit, How the symptom change?  □ markedly improved □ improved □ not change □ Worse □ Worst | | | |

Modified from Häggström’s et al (2008) protocol^1^

**Supplementary Table 3.** The number of dogs receiving medication from both groups is displayed before recruitment into the study and each presentation.

| Medication | Number of dogs | | | | | | | | | |
| --- | --- | --- | --- | --- | --- | --- | --- | --- | --- | --- |
|  | EAB-277 | | | | | Placebo | | | | |
|  | Before  visit | Day 0 | Day 14 | Day 28 | Day 56 | Before  visit | Day 0 | Day 14 | Day 28 | Day 56 |
| Per oral |  |  |  |  |  |  |  |  |  |  |
| Doxophylline | - | - | - | - | - | 1 | 1 | - | - | - |
| Theophylline | 2 | - | - | - | 1 | 1 | - | - | - | - |
| Aminophylline | 4 | 4 | - | - | - | 3 | 3 | 3 | - | - |
| Carbocysteine | 2 | 4 | 2 | 2 | 2 | 4 | 4 | 3 | 2 | 1 |
| Acetylcysteine | - | - | - | - | - | 2 | - | 1 | 1 | - |
| Codeine | - | - | - | - | - | - | 1 | 1 | - | - |
| Prednisolone | 5 | - | - | - | - | 5 | - | - | 1 | - |
| Hydroxyzine | 1 | - | - | - | - | - | - | - | - | - |
| Amoxycillin | - | - | - | - | - | 1 | - | - | - | - |
| Nebulization |  |  |  |  |  |  |  |  |  |  |
| NSS | 3 | 2 | 4 | 3 | 3 | 2 | 2 | 4 | 5 | 3 |
| Salbutamol | 4 | 3 | 1 | 2 | 2 | 3 | 1 | 1 | 2 | 2 |
| Fluticasone | 2 | 1 | 1 | 1 | 1 | 4 | - | 2 | 3 | 1 |
| Budesonide | 1 | 3 | 3 | 3 | 3 | - | - | - | - | - |

|  | EAB 277 | | | | | Placebo | | | | |
| --- | --- | --- | --- | --- | --- | --- | --- | --- | --- | --- |
|  | Before day 0 | Day 0 | Day 14 | Day 28 | Day 56 | Before day 0 | Day 0 | Day 14 | Day 28 | Day 56 |
| No medication | 12 | 12 | 14 | 14 | 12 | 12 | 11 | 12 | 14 | 17 |
| Mo, - | - | 1 | 2 | 2 | 3 | 2 | 2 | 2 | 2 | - |
| Mo, Nn | - | - | 1 | - | - | - | - | - | - | - |
| Mo, Gn | 1 | 1 |  | - | - | - | - | - | - | - |
| Mo, NnGn | - | 1 | 1 | 1 | - | - | - | - | 2 | - |
| Bo, - | 2 | 1 | - | 1 | - | - | - | - | - | - |
| Bo, Nn | 1 | - | - | - | - | - | - | - | - | - |
| Bo, Gn | 1 | - | - | - | 1 | - | - | - | - | - |
| Bo, NnBnGn | 1 | 1 | - | - | - | - | - | - | - | - |
| MoBo, - | 1 | - | - | - | - | 2 | 3 | - | - | - |
| MoBo, Nn | - | 1 | - | - | - | 1 | 1 | - | - | - |
| MoBo, Gn | - | - | - | - | - | - | - | - | - | - |
| MoBo, NnGn | - | - | - | - | - | - | - | 2 | - | - |
| MoBo, BnGn | 1 | 1 | - | - | - | 1 | - | - | - | - |
| MoBo, NnBnGn | - | - | - | - | - | 2 | - | - | - | - |
| MoBoAo, - | - | - | - | - | - | - | 1 | 1 | - | - |
| -, Nn | - | - | 1 | 1 | 1 | - | 1 | 1 | 2 | 2 |
| -, Gn | 1 | 1 | 1 | 1 | 1 | 1 |  | 1 | 1 | 1 |
| -, NnGn | - | - | 1 | 1 | 1 | - | - | - | - | - |
| -, BnGn | 1 | 1 | 1 | 1 | 1 | - | - | - | - | - |

**Supplementary Table 4.**The number of dogs receiving medication from both groups is displayed before recruitment into the study and each presentation, separated by medication group and route of administration.

Abbreviations: Mo= Mucolytic drugs (per oral route), Bo= Bronchodilator drugs (per oral route), Ao= Antitussive drugs (per oral route), Nn = Normal saline (administration with a nebulizer), Gn= Glucocorticoid drugs (administration with a nebulizer), Bn= Bronchodilator drugs (administration with a nebulizer)

| Variables/visits | Group 1 EAB-277  (n=21) | Group 2 Placebo  (n=20) | P  between group | P model  repeated measured | P different between visits within subjects of group 1 | | | P model  repeated measured | P different between visits within subjects of group 2 | | |
| --- | --- | --- | --- | --- | --- | --- | --- | --- | --- | --- | --- |
|  |  |  | 1 vs 2 | group 1 | 1 vs 2 | 1 vs 4 | 2vs 4 | group 2 | 1 vs 2 | 1 vs 4 | 2vs 4 |
| Cervical region |  |  |  | 0.097 | 0.016 | 0.013 | 0.455 | 0.058 | 0.296 | 0.017 | 0.167 |
| 1st visit | 12.58(6.54,24.40) | 12.39(7.15,17.57) | 0.481 |  |  |  |  |  |  |  |  |
| 2nd visit | 7.88(3.66,14.21) | 9.98(4.98,14.14) | 0.303 |  |  |  |  |  |  |  |  |
| 4th visit | 7.17(3.85,10.40) | 8.27(3.65,12.23) | 0.549 |  |  |  |  |  |  |  |  |
| Thoracic inlet region |  |  |  | 0.047 | 0.003 | 0.149 | 0.192 | 0.522 | 0.145 | 0.156 | 0.654 |
| 1st visit | 15.59(10.35,24.03) | 15.87(9.15,20.67) | 0.784 |  |  |  |  |  |  |  |  |
| 2nd visit | 11.52(7.37,13.49) | 12.03(9.03,19.78) | 0.257 |  |  |  |  |  |  |  |  |
| 4th visit | 12.61(8.79,16.95) | 10.29(8.54,17.26) | 0.449 |  |  |  |  |  |  |  |  |
| Intra-thoracic region |  |  |  | 0.097 | 0.009 | 0.016 | 0.848 | 0.091 | 0.108 | 0.191 | 0.911 |
| 1st visit | 17.09(10.80,28.25) | 15.00(10.97,20.15) | 0.584 |  |  |  |  |  |  |  |  |
| 2nd visit | 10.02(8.23,19.01) | 13.57(6.67,19.09) | 0.715 |  |  |  |  |  |  |  |  |
| 4th visit | 11.89(8.68,17.87) | 11.44(7.58,22.04) | 0.979 |  |  |  |  |  |  |  |  |
| Carina region |  |  |  | 0.467 | 0.614 | 0.159 | 0.394 | 0.951 | 0.970 | 0.455 | 0.370 |
| 1st visit | 20.47(14.31,37.43) | 20.99(14.01,28.05) | 0.845 |  |  |  |  |  |  |  |  |
| 2nd visit | 20.81(13.49,27.70) | 18.43(13.93,31.83) | 1.000 |  |  |  |  |  |  |  |  |
| 4th visit | 18.50(14.60,24.50) | 17.23(11.18,26.99) | 0.835 |  |  |  |  |  |  |  |  |

**Supplementary Table 5.** Comparisons of tracheal height percentage difference obtained by fluoroscopy between full inspiration and expiration phrase divided into 4 regions for each visit between group 1 (EAB-277) and group 2 (placebo). Comparisons between the group were tested Mann-Whitney U test. Comparisons between visits within subjects in each group were tested by Friedman with pairwise Wilcoxon signed-rank test. (Visit 1 = Day 0, Visit 2 = Day 14, and Visit 4 = Day 56 /end of treatment)

**Supplementary Table 6.** Comparisons of ratio between greatest tracheal height and T1 from radiography divided into 4 regions for each visit between group 1 (EAB-277) and group 2 (placebo). Comparisons between group was tested Mann-Whitney U test. Comparisons between visits within subjects each group were tested by Friedman with pairwise Wilcoxon signed-rank test. (Visit 1 = Day 0, Visit 2 = Day 14, and Visit 4 = Day 56 /end of treatment)

| Variables/visits | Group 1 EAB-277  (n=21) | Group 2 Placebo  (n=20) | P between group 1 vs 2 | P model repeated measured | P different between visits within subjects of group 1 | | | P model  repeated measured | P different between visits within subjects of group 2 | | |
| --- | --- | --- | --- | --- | --- | --- | --- | --- | --- | --- | --- |
|  |  |  |  | group 1 | 1 vs 2 | 1 vs 4 | 2vs 4 | group 2 | 1 vs 2 | 1 vs 4 | 2vs 4 |
| Cervical region |  |  |  | 0.129 | 0.018 | 0.702 | 0.322 | 0.186 | 0.038 | 0.161 | 0.211 |
| 1st visit | 1.85(1.56,2.20) | 1.79(1.22,2.22) | 0.426 |  |  |  |  |  |  |  |  |
| 2nd visit | 2.07(1.52,2.34) | 1.89(1.52,2.59) | 0.705 |  |  |  |  |  |  |  |  |
| 4th visit | 1.92(1.44,2.27) | 1.92(1.40,2.22) | 0.855 |  |  |  |  |  |  |  |  |
| Thoracic inlet region |  |  |  | 0.129 | 0.046 | 0.715 | 0.046 | 0.705 | 0.145 | 0.204 | 0.411 |
| 1st visit | 1.72(1.37,2.26) | 1.43(1.18,1.89) | 0.050 |  |  |  |  |  |  |  |  |
| 2nd visit | 2.03 (1.51,2.46) | 1.38(1.08,2.17) | 0.032 |  |  |  |  |  |  |  |  |
| 4th visit | 1.65 (1.40,2.27) | 1.41(1.25,1.89) | 0.130 |  |  |  |  |  |  |  |  |
| Intra-thoracic region |  |  |  | 0.013 | 0.002 | 0.170 | 0.085 | 0.047 | 0.044 | 0.654 | 0.033 |
| 1st visit | 1.17(1.05,1.46) | 1.32(0.99,1.48) | 0.979 |  |  |  |  |  |  |  |  |
| 2nd visit | 1.44(1.25,1.85) | 1.38(1.12,1.70) | 0.620 |  |  |  |  |  |  |  |  |
| 4th visit | 1.33 (1.13,1.58) | 1.25(1.02,1.48) | 0.657 |  |  |  |  |  |  |  |  |
| Carina region |  |  |  | 0.097 | 0.122 | 0.305 | 0.063 | 0.387 | 0.117 | 0.391 | 0.391 |
| 1st visit | 1.26 (1.00,1.64) | 1.15(1.04,1.45) | 0.531 |  |  |  |  |  |  |  |  |
| 2nd visit | 1.30 (1.06,1.69) | 1.39 (1.06,1.79) | 0.855 |  |  |  |  |  |  |  |  |
| 4th visit | 1.32 (0.98,1.42) | 1.31(1.00,1.51) | 0.602 |  |  |  |  |  |  |  |  |

**Supplementary Table 7.** Comparisons of ratio between shortest tracheal height and T1 from radiography divided into 4 regions for each visit between group 1 (EAB-277) and group 2 (placebo). The variables were tested by independent T-test for normally distributed variables and Mann-Whitney U test for non-normally distributed variables. Comparisons between visits within subjects each group were tested by repeated measured general linear model with pairwise Bonferroni’s test for normally distributed variables or Friedman with pairwise Wilcoxon signed-rank test for non-normal distributed variables. (Visit 1 = Day 0, Visit 2 = Day 14, and Visit 4 = Day 56 /end of treatment)

| Variables/visits | Group 1 EAB-277  (n=21) | Group 2 Placebo  (n=20) | P between group 1 vs 2 | P model repeated measured | P different between visits within subjects of group 1 | | | P model  repeated measured | P different between visits within subjects of group 2 | | |
| --- | --- | --- | --- | --- | --- | --- | --- | --- | --- | --- | --- |
|  |  |  |  | group 1 | 1 vs 2 | 1 vs 4 | 2vs 4 | group 2 | 1 vs 2 | 1 vs 4 | 2vs 4 |
| Cervical region |  |  |  | 0.312 | 0.038 | 1.000 | 0.886 | 0.131 | 0.243 | 1.000 | 0.640 |
| 1st visit | 1.73±0.47 | 1.63±0.55 | 0.568 |  |  |  |  |  |  |  |  |
| 2nd visit | 1.94±0.60 | 1.85±0.73 | 0.687 |  |  |  |  |  |  |  |  |
| 4th visit | 1.67±0.55 | 1.73±0.63 | 0.753 |  |  |  |  |  |  |  |  |
| Thoracic inlet region |  |  |  | 0.174 | 0.015 | 0.196 | 0.435 | 0.705 | 0.351 | 0.478 | 0.332 |
| 1st visit | 1.57(1.27,1.87) | 1.31(1.01,1.58) | 0.039 |  |  |  |  |  |  |  |  |
| 2nd visit | 1.60(1.36,2.16) | 1.31(1.02,1.76) | 0.019 |  |  |  |  |  |  |  |  |
| 4th visit | 1.57(1.25,1.94) | 1.31(0.99,1.66) | 0.086 |  |  |  |  |  |  |  |  |
| Intra-thoracic region |  |  |  | 0.144 | 0.062 | 0.569 | 0.435 | 0.056 | 0.028 | 0.287 | 0.204 |
| 1st visit | 1.09(0.99,1.25) | 1.12(0.87,1.27) | 0.610 |  |  |  |  |  |  |  |  |
| 2nd visit | 1.19(1.04,1.39) | 1.06(0.93,1.55) | 0.593 |  |  |  |  |  |  |  |  |
| 4th visit | 1.10(1.07,1.35) | 1.09(0.90,1.26) | 0.383 |  |  |  |  |  |  |  |  |
| Carina region |  |  |  | 0.611 | 1.000 | 1.000 | 1.000 | 0.066 | 0.079 | 0.436 | 1.000 |
| 1st visit | 1.09±0.29 | 1.06±0.32 | 0.776 |  |  |  |  |  |  |  |  |
| 2nd visit | 1.14±0.36 | 1.21±0.34 | 0.506 |  |  |  |  |  |  |  |  |
| 4th visit | 1.10±0.24 | 1.13±0.39 | 0.739 |  |  |  |  |  |  |  |  |

**Supplementary Table** **8.** Comparisons of ratio between greatest tracheal height and proximal third of 3rd rib from radiography divided into 4 regions for each visit between group 1 (EAB-277) and group 2 (placebo). Comparisons between group was tested Mann-Whitney U test. Comparisons between visits within subjects each group were tested by Friedman with pairwise Wilcoxon signed-rank test. (Visit 1 = Day 0, Visit 2 = Day 14, and Visit 4 = Day 56 /end of treatment)

| Variables/visits | Group 1 EAB-277  (n=21) | Group 2 Placebo  (n=20) | P between group  1 vs 2 | P model repeated measured | P different between visits within subjects of group 1 | | | P model  repeated measured | P different between visits within subjects of group 2 | | |
| --- | --- | --- | --- | --- | --- | --- | --- | --- | --- | --- | --- |
|  |  |  |  | group 1 | 1 vs 2 | 1 vs 4 | 2vs 4 | group 2 | 1 vs 2 | 1 vs 4 | 2vs 4 |
| Cervical region |  |  |  | 0.102 | 0.052 | 0.664 | 0.068 | 0.449 | 0.218 | 0.057 | 0.765 |
| 1st visit | 3.44(2.76,4.53) | 3.26(2.75,4.31) | 0.411 |  |  |  |  |  |  |  |  |
| 2nd visit | 4.29(2.82,5.1) | 3.88(2.55,4.56) | 0.540 |  |  |  |  |  |  |  |  |
| 4th visit | 3.28(2.42,4.54) | 3.58(2.83,4.67) | 0.368 |  |  |  |  |  |  |  |  |
| Thoracic inlet region |  |  |  | 0.023 | 0.079 | 0.520 | 0.009 | 0.350 | 0.351 | 0.218 | 0.852 |
| 1st visit | 3.10 (2.22,4.61) | 2.94(1.90,3.51) | 0.230 |  |  |  |  |  |  |  |  |
| 2nd visit | 3.94 (2.64,4.98) | 2.93(2.15,3.72) | 0.022 |  |  |  |  |  |  |  |  |
| 4th visit | 3.04 (2.09,4.72) | 3.03(2.34,3.81) | 0.715 |  |  |  |  |  |  |  |  |
| Intra-thoracic region |  |  | 0.715 | 0.027 | 0.002 | 0.274 | 0.035 | 0.705 | 0.247 | 0.263 | 0.654 |
| 1st visit | 2.35(1.87,2.79) | 2.43(1.97,2.88) | 0.715 |  |  |  |  |  |  |  |  |
| 2nd visit | 2.72 (2.20,3.50) | 2.62(2.03,3.18) | 0.566 |  |  |  |  |  |  |  |  |
| 4th visit | 2.24(1.89,3.02) | 2.34(1.92,3.08) | 0.835 |  |  |  |  |  |  |  |  |
| Carina region |  |  |  | 0.047 | 0.046 | 0.140 | 0.035 | 0.350 | 0.191 | 0.156 | 0.794 |
| 1st visit | 2.33(1.87,2.80) | 2.27(1.68,2.91) | 0.754 |  |  |  |  |  |  |  |  |
| 2nd visit | 2.56(2.01,3.04) | 2.72(2.06,3.38) | 0.958 |  |  |  |  |  |  |  |  |
| 4th visit | 2.21(1.77,2.79) | 2.32(2.01,3.02) | 0.309 |  |  |  |  |  |  |  |  |

**Supplementary Table 9.** Comparisons of ratio between shortest tracheal height and proximal third of 3^rd^ rib from radiography divided into 4 regions for each visit between group 1 (EAB-277) and group 2 (placebo). The variables were tested by independent T-test for normally distributed variables and Mann-Whitney U test for non-normally. distributed variables. Comparisons between visits within subjects each group were tested by repeated measured general linear model with pairwise Bonferroni’s test for normally distributed variables or Friedman with pairwise Wilcoxon signed-rank test for non-normal distributed variables. (Visit 1 = Day 0, Visit 2 = Day 14, and Visit 4 = Day 56 /end of treatment)

| Variables/visits | Group 1 EAB-277  (n=21) | Group 2 Placebo  (n=20) | P between group 1 vs 2 | P model repeated measured | P different between visits within subjects of group 1 | | | P model  repeated measured | P different between visits within subjects of group 2 | | |
| --- | --- | --- | --- | --- | --- | --- | --- | --- | --- | --- | --- |
|  |  |  |  | group 1 | 1 vs 2 | 1 vs 4 | 2vs 4 | group 2 | 1 vs 2 | 1 vs 4 | 2vs 4 |
| Cervical region |  |  |  | 0.174 | 0.009 | 1.000 | 0.480 | 0.584 | 1.000 | 0.927 | 1.000 |
| 1st visit | 3.25±0.87 | 3.09±1.03 | 0.628 |  |  |  |  |  |  |  |  |
| 2nd visit | 3.80±1.27 | 3.32±1.21 | 0.246 |  |  |  |  |  |  |  |  |
| 4th visit | 3.08±1.30 | 3.39±1.33 | 0.459 |  |  |  |  |  |  |  |  |
| Thoracic inlet region |  |  |  | 0.155 | 0.015 | 0.836 | 0.102 | 0.951 | 0.823 | 0.411 | 0.970 |
| 1st visit | 2.81(2.18,4.15) | 2.49(2.00,2.93) | 0.133 |  |  |  |  |  |  |  |  |
| 2nd visit | 3.31(2.51,4.49) | 2.62(1.77,3.29) | 0.030 |  |  |  |  |  |  |  |  |
| 4th visit | 2.76(2.00,4.07) | 2.46(1.94,3.42) | 0.414 |  |  |  |  |  |  |  |  |
| Intra-thoracic region |  |  |  | 0.327 | 0.093 | 0.717 | 0.084 | 0.327 | 0.309 | 0.246 | 0.911 |
| 1st visit | 2.11(1.86,2.44) | 2.09(1.78,2.48) | 0.892 |  |  |  |  |  |  |  |  |
| 2nd visit | 2.27(1.95,2.75) | 2.14(1.73,2.98) | 0.573 |  |  |  |  |  |  |  |  |
| 4th visit | 2.05(1.74,2.58) | 2.01(1.73,2.54) | 0.989 |  |  |  |  |  |  |  |  |
| Carina region |  |  |  | 0.434 | 1.000 | 1.000 | 0.983 | 0.272 | 1.000 | 0.171 | 1.000 |
| 1st visit | 2.01±0.50 | 2.06±0.73 | 0.845 |  |  |  |  |  |  |  |  |
| 2nd visit | 2.18±0.73 | 2.24±0.75 | 0.810 |  |  |  |  |  |  |  |  |
| 4th visit | 2.03±0.65 | 2.23±0.90 | 0.435 |  |  |  |  |  |  |  |  |

**Supplementary Table 10.** Comparisons of greatest tracheal height ratio minus shortest tracheal height ratio divided by greatest tracheal height ratio calculated in percent at T1 from radiography divided into 4 regions for each visit between group 1 (EAB-277) and group 2 (placebo). Comparisons between group was tested by Mann-Whitney U test. Comparisons between visits within subjects each group were tested by Friedman with pairwise Wilcoxon signed-rank test. (Visit 1 = Day 0, Visit 2 = Day 14, and Visit 4 = Day 56 /end of treatment)

| Variables/visits | Group 1 EAB-277  (n=21) | Group 2 Placebo  (n=20) | P between group 1 vs 2 | P model repeated measured | P different between visits within subjects of group 1 | | | P model  repeated measured | P different between visits within subjects of group 2 | | |
| --- | --- | --- | --- | --- | --- | --- | --- | --- | --- | --- | --- |
|  |  |  |  | group 1 | 1 vs 2 | 1 vs 4 | 2vs 4 | group 2 | 1 vs 2 | 1 vs 4 | 2vs 4 |
| Cervical region |  |  |  | 0.420 | 0.255 | 0.756 | 0.255 | 0.672 | 0.535 | 0.569 | 0.948 |
| 1st visit | 12.29(8.35,15.48) | 8.82(3.80,12.66) | 0.209 |  |  |  |  |  |  |  |  |
| 2nd visit | 6.54(1.98,12.23) | 6.94(3.35,17.87) | 0.532 |  |  |  |  |  |  |  |  |
| 4th visit | 12.95(4.78,17.75) | 6.24(5.02,16.11) | 0.413 |  |  |  |  |  |  |  |  |
| Thoracic inlet region |  |  |  | 0.074 | 0.569 | 0.196 | 0.098 | 0.790 | 0.407 | 0.906 | 0.391 |
| 1st visit | 14.79(7.02,19.60) | 15.54(6.07,23.80) | 0.823 |  |  |  |  |  |  |  |  |
| 2nd visit | 11.33(9.33,17.08) | 6.46 (3.69,20.30) | 0.190 |  |  |  |  |  |  |  |  |
| 4th visit | 9.03(2.83,20.73) | 11.53(4.59,20.96) | 0.613 |  |  |  |  |  |  |  |  |
| Intra-thoracic region |  |  |  | 0.397 | 0.363 | 0.807 | 0.044 | 0.420 | 0.733 | 0.460 | 0.526 |
| 1st visit | 9.55(4.63,20.53) | 13.67(8.91,17.02) | 0.330 |  |  |  |  |  |  |  |  |
| 2nd visit | 16.27(10.96,20.84) | 13.65(8.04,19.84) | 0.559 |  |  |  |  |  |  |  |  |
| 4th visit | 8.26(3.70,17.48) | 10.69(6.94,18.69) | 0.384 |  |  |  |  |  |  |  |  |
| Carina region |  |  |  | 0.662 | 0.472 | 0.586 | 0.157 | 0.819 | 0.427 | 0.215 | 0.904 |
| 1st visit | 11.42(7.08,18.67) | 17.70(9.40,22.93) | 0.370 |  |  |  |  |  |  |  |  |
| 2nd visit | 16.15(6.67,21.71) | 11.46(4.58,20.27) | 0.715 |  |  |  |  |  |  |  |  |
| 4th visit | 11.04(6.61,18.97) | 14.56(3.80,20.66) | 0.808 |  |  |  |  |  |  |  |  |

**Supplementary Table 11.** Comparisons of greatest tracheal height ratio minus shortest tracheal height ratio divided by greatest tracheal height ratio calculated in percent at proximal third of 3^rd^ rib from radiography divided into 4 regions for each visit between group 1 (EAB-277) and group 2 (placebo). Comparisons between group was tested by Mann-Whitney U test. Comparisons between visits within subjects each group were tested by Friedman with pairwise Wilcoxon signed-rank test. (Visit 1 = Day 0, Visit 2 = Day 14, and Visit 4 = Day 56 /end of treatment)

| Variables/visits | Group 1 EAB-277  (n=21) | Group 2 Placebo  (n=20) | P between group 1 vs 2 | P model repeated measured | P different between visits within subjects of group 1 | | | P model  repeated measured | P different between visits within subjects of group 2 | | |
| --- | --- | --- | --- | --- | --- | --- | --- | --- | --- | --- | --- |
|  |  |  |  | group 1 | 1 vs 2 | 1 vs 4 | 2vs 4 | group 2 | 1 vs 2 | 1 vs 4 | 2vs 4 |
| Cervical region |  |  |  | 0.420 | 0.326 | 0.756 | 0.234 | 0.838 | 0.523 | 0.687 | 1.000 |
| 1st visit | 12.21(8.31,15.30) | 8.87(3.94,12.48) | 0.228 |  |  |  |  |  |  |  |  |
| 2nd visit | 6.35(1.83,12.26) | 6.78(3.52,17.82) | 0.522 |  |  |  |  |  |  |  |  |
| 4th visit | 12.94(4.73,17.87) | 6.06(4.83,16.01) | 0.299 |  |  |  |  |  |  |  |  |
| Thoracic inlet region |  |  |  | 0.125 | 0.307 | 0.140 | 0.173 | 0.790 | 0.332 | 0.906 | 0.411 |
| 1st visit | 17.58(8.12,23.05) | 15.41(6.07,23.98) | 0.770 |  |  |  |  |  |  |  |  |
| 2nd visit | 11.35(9.44,16.80) | 6.25(3.58,20.17) | 0.180 |  |  |  |  |  |  |  |  |
| 4th visit | 9.54(2.91,20.96) | 11.46(4.56,20.95) | 0.653 |  |  |  |  |  |  |  |  |
| Intra-thoracic region |  |  |  | 0.282 | 0.234 | 0.532 | 0.025 | 0.247 | 0.733 | 0.394 | 0.478 |
| 1st visit | 8.42(2.91,19.53) | 13.49(8.61,17.31) | 0.236 |  |  |  |  |  |  |  |  |
| 2nd visit | 16.20(10.95,20.83) | 14.04(8.06,19.70) | 0.501 |  |  |  |  |  |  |  |  |
| 4th visit | 7.99(3.62,16.90) | 10.37(6.92,18.77) | 0.330 |  |  |  |  |  |  |  |  |
| Carina region |  |  |  | 0.829 | 0.795 | 0.492 | 0.266 | 0.838 | 0.287 | 0.332 | 0.970 |
| 1st visit | 11.32(7.38,18.87) | 15.00(7.49,22.29) | 0.680 |  |  |  |  |  |  |  |  |
| 2nd visit | 15.31(6.22,19.84) | 11.72(4.41,19.32) | 0.726 |  |  |  |  |  |  |  |  |
| 4th visit | 10.96(6.53,19.06) | 14.66(3.17,20.66) | 0.766 |  |  |  |  |  |  |  |  |

**Supplementary Table 12.** Comparison of percent change of tracheal diameter in both groups between fluoroscopic and radiographic results (both indexes) separated in each region and day of visit Comparisons between two methods were tested by Wilcoxon signed-rank test.

| Region | A. Fluoroscopy | B. Radiography   ( T1) | C. Radiography   (Rib 3) | A vs B | A vs C | B vs C |
| --- | --- | --- | --- | --- | --- | --- |
| Day 0 |  |  |  |  |  |  |
| Cervical | 13.12(7.63-19.86) | 10.38(4.73-13.42) | 10.52(4.88-13.23) | 0.138 | 0.140 | 0.453 |
| Thoracic inlet | 16.53(10.69-21.89) | 15.17(7.43-21.23) | 16.50(8.17-23.26) | 0.948 | 0.568 | 0.865 |
| Intra-thoracic | 17.47(10.90-22.43) | 10.00(5.75-16.55) | 9.95(5.81-16.99) | 0.021 | 0.023 | 0.063 |
| Carina | 25.27(14.62-34.40) | 11.59(7.14-20.31) | 11.52(7.42-20.65) | 0.003 | 0.003 | 0.063 |
| Day 14 |  |  |  |  |  |  |
| Cervical | 8.04(4.15-14.21) | 6.54(2.40-14.77) | 6.42(2.51-14.58) | 0.839 | 0.839 | 0.177 |
| Thoracic inlet | 11.34 (8.43-16.20) | 10.05(4.85-17.99) | 10.05(4.83-17.65) | 0.934 | 0.946 | 0.322 |
| Intra-thoracic | 11.89(7.64-19.08) | 15.19(9.87-20.32) | 15.52(10.39-20.20) | 0.300 | 0.267 | 0.076 |
| Carina | 20.10(13.74-28.40) | 13.86(5.26-20.27) | 12.81(4.83-19.35) | 0.016 | 0.004 | 0.808 |
| Day 56 |  |  |  |  |  |  |
| Cervical | 7.21(3.60-10.36) | 10.87(4.63-15.54) | 10.69(4.54-15.82) | 0.062 | 0.058 | 0.425 |
| Thoracic inlet | 10.40(8.56-15.78) | 10.64(3.31-20.39) | 10.59(3.29-20.41) | 0.902 | 0.948 | 0.586 |
| Intra-thoracic | 11.44(7.86-16.77) | 9.60(5.21-17.13) | 9.42(5.01-16.90) | 0.455 | 0.438 | 0.498 |
| Carina | 17.23(12.86-24.99) | 11.53(5.11-19.17) | 11.38(5.00-19.33) | 0.000 | 0.000 | 0.484 |

**Supplementary Table 13.** Comparisons of heamatology and biochemistry profiles for each visit between group 1 (EAB-277) and group 2 (placebo). The variables were tested by independent T-test for normally distributed variables and Mann-Whitney U test for non-normally distributed variables. Comparisons between visits within subjects each group were tested by repeated measured general linear model with pairwise Bonferroni’s test for normally distributed variables or Friedman with pairwise Wilcoxon signed-rank test for non-normal distributed variables. (Visit 1 = Day 0, Visit 2 = Day 14, Visit 3 = Day 28, and Visit 4 = Day 56 /end of treatment)

Abbreviations WBC, White blood cell count; NEU, neutrophil count; LYM, lymphocyte count; NEU/LYM, neutrophil to lymphocyte count ratio; MONO, monocyte count; EOS, eosinophil count; RBC, red blood cell count; Hb, hemoglobin; Hct, hematocrit; MCV, mean corpuscular volume; MCH, mean corpuscular hemoglobin; MCHC, mean corpuscular hemoglobin concentration; PLT, platelet; RDW, Red blood cell distribution width; ALB, albumin; ALT, alanine aminotransferase; ALP, alkaline phosphatase; BUN, blood urea nitrogen; crea, creatinine; TP, total protein; GLO, globulin; A:G ratio, albumin to globulin ratio; PP, plasma protein

| Variables/visits | Group 1 EAB-277  (n=21) | Group 2 Placebo  (n=20) | P  between group | P model  repeated measured | P different between visits within subjects of group 1 | | | | | | P model  repeated measured | P different between visits within subjects of group 2 | | | | | |
| --- | --- | --- | --- | --- | --- | --- | --- | --- | --- | --- | --- | --- | --- | --- | --- | --- | --- |
|  |  |  |  | group 1 | 1 vs 2 | 1 vs 3 | 1 vs 4 | 2 vs 3 | 2 vs 4 | 3 vs 4 | group 2 | 1 vs 2 | 1 vs 3 | 1 vs 4 | 2 vs 3 | 2 vs 4 | 3 vs 4 |
| WBC |  |  |  | 0.596 | 0.212 | 0.445 | 0.215 | 0.856 | 0.379 | 0.148 | 0.031 | 0.020 | 0.037 | 0.086 | 0.778 | 0.904 | 0.601 |
| Visit 1 | 11.6(10.0,14.1) | 13.8(10.5,18.6) | 0.088 |  |  |  |  |  |  |  |  |  |  |  |  |  |  |
| Visit 2 | 12.2(9.4,13.4) | 11.5(8.6,13.4) | 0.786 |  |  |  |  |  |  |  |  |  |  |  |  |  |  |
| Visit 3 | 11.8(10.0,13.4) | 11.2(9.1,15.4) | 0.925 |  |  |  |  |  |  |  |  |  |  |  |  |  |  |
| Visit 4 | 10.8(8.8,12.6) | 10.4(8.4,14.9) | 0.501 |  |  |  |  |  |  |  |  |  |  |  |  |  |  |
| NEU |  |  |  | 0.258 | 0.260 | 0.334 | 0.121 | 0.809 | 0.569 | 0.148 | 0.029 | 0.022 | 0.040 | 0.086 | 0.748 | 0.904 | 0.526 |
| Visit 1 | 8.5(7.1,11.0) | 9.5(7.8,14.2) | 0.099 |  |  |  |  |  |  |  |  |  |  |  |  |  |  |
| Visit 2 | 9.0(6.5,9.7) | 8.0(6.2,9.4) | 0.616 |  |  |  |  |  |  |  |  |  |  |  |  |  |  |
| Visit 3 | 8.8(7.1,10.4) | 8.0(6.5,11.8) | 0.665 |  |  |  |  |  |  |  |  |  |  |  |  |  |  |
| Visit 4 | 7.7(5.2,9.2) | 7.8(6.1,10.1) | 0.483 |  |  |  |  |  |  |  |  |  |  |  |  |  |  |
| LYM |  |  |  | 0.419 | 0.601 | 0.295 | 0.134 | 0.872 | 0.255 | 0.379 | 0.281 | 0.033 | 0.167 | 0.179 | 0.872 | 0.809 | 0.852 |
| Visit 1 | 2.4(2.0,2.8) | 2.6(2.0,3.3) | 0.317 |  |  |  |  |  |  |  |  |  |  |  |  |  |  |
| Visit 2 | 2.2 (1.7,2.6) | 2.2(1.7,2.8) | 0.755 |  |  |  |  |  |  |  |  |  |  |  |  |  |  |
| Visit 3 | 2.1(1.9,2.7) | 2.3(2.0,2.9) | 0.273 |  |  |  |  |  |  |  |  |  |  |  |  |  |  |
| Visit 4 | 1.9(1.2,2.8) | 2.2(1.5,3.0) | 0.242 |  |  |  |  |  |  |  |  |  |  |  |  |  |  |
| NEU/LYM |  |  |  | 0.859 | 0.845 | 0.546 | 0.605 | 0.687 | 0.394 | 0.796 | 0.454 | 0.601 | 0.391 | 0.391 | 0.936 | 1.000 | 0.881 |
| Visit 1 | 3.9(3.1,4.8) | 4.1 (3.5,4.8) | 0.402 |  |  |  |  |  |  |  |  |  |  |  |  |  |  |
| Visit 2 | 3.8(2.9,4.9) | 3.8(3.0,5.1) | 0.768 |  |  |  |  |  |  |  |  |  |  |  |  |  |  |
| Visit 3 | 3.7(3.0,5.2 | 3.7(3.1,5.0) | 0.808 |  |  |  |  |  |  |  |  |  |  |  |  |  |  |
| Visit 4 | 3.9(2.9,6.3) | 3.7(3.1,4.7) | 0.561 |  |  |  |  |  |  |  |  |  |  |  |  |  |  |
|  |  |  |  |  |  | | | | | |  |  | | | | | |
|  |  |  |  |  |  | | | | | |  |  | | | | | |
|  |  |  |  |  |  | | | | | |  |  | | | | | |
| Variables/visits | Group 1 EAB-277  (n=21) | Group 2 Placebo  (n=20) | P  between group | P model  repeated measured | P different between visits within subjects of group 1 | | | | | | P model  repeated measured | P different between visits within subjects of group 2 | | | | | |
|  |  |  |  | group 1 | 1 vs 2 | 1 vs 3 | 1 vs 4 | 2 vs 3 | 2 vs 4 | 3 vs 4 | group 2 | 1 vs 2 | 1 vs 3 | 1 vs 4 | 2 vs 3 | 2 vs 4 | 3 vs 4 |
| MONO |  |  |  | 0.414 | 0.862 | 0.765 | 0.286 | 0.550 | 0.396 | 0.193 | 0.133 | 0.295 | 0.411 | 0.296 | 0.227 | 0.546 | 0.296 |
| Visit 1 | 0.6(0.4,0.7) | 0.7(0.40.9) | 0.183 |  |  |  |  |  |  |  |  |  |  |  |  |  |  |
| Visit 2 | 0.5(0.4,0.8) | 0.5(0.3,0.8) | 0.882 |  |  |  |  |  |  |  |  |  |  |  |  |  |  |
| Visit 3 | 0.5(0.4,0.9) | 0.6(0.4,0.8) | 0.808 |  |  |  |  |  |  |  |  |  |  |  |  |  |  |
| Visit 4 | 0.4(0.2,0.8) | 0.5(0.3,0.8) | 0.179 |  |  |  |  |  |  |  |  |  |  |  |  |  |  |
| EOS |  |  |  | 0.608 | 0.445 | 0.184 | 0.959 | 0.586 | 0.255 | 0.177 | 0.382 | 0.616 | 0.681 | 0.352 | 0.445 | 0.446 | 0.126 |
| Visit 1 | 0.2(0.1,0.3) | 0.2(0.0,0.5) | 0.279 |  |  |  |  |  |  |  |  |  |  |  |  |  |  |
| Visit 2 | 0.2(0.1,0.4) | 0.2(0.1,0.5) | 0.446 |  |  |  |  |  |  |  |  |  |  |  |  |  |  |
| Visit 3 | 0.3(0.1,0.5) | 0.3(0.1,0.4) | 0.341 |  |  |  |  |  |  |  |  |  |  |  |  |  |  |
| Visit 4 | 0.2(0.1,0.4) | 0.2(0.0,0.3) | 0.148 |  |  |  |  |  |  |  |  |  |  |  |  |  |  |
| RBC |  |  |  | 0.194 | 0.316 | 0.212 | 0.326 | 0.251 | 0.211 | 0.836 | 0.601 | 0.809 | 0.179 | 0.332 | 0.457 | 0.587 | 0.695 |
| Visit 1 | 6.76(5.74,7.23) | 6.33(5.65,7.22) | 0.665 |  |  |  |  |  |  |  |  |  |  |  |  |  |  |
| Visit 2 | 6.73(6.05,7.31) | 6.64(6.04,7.07) | 0.673 |  |  |  |  |  |  |  |  |  |  |  |  |  |  |
| Visit 3 | 6.61(6.10,7.612) | 6.72(6.16,7.07) | 0.579 |  |  |  |  |  |  |  |  |  |  |  |  |  |  |
| Visit 4 | 6.72(6.30,7.43) | 6.85(6.23,7.35) | 0.552 |  |  |  |  |  |  |  |  |  |  |  |  |  |  |
| Hb |  |  |  | 0.204 | 0.268 | 0.184 | 0.698 | 0.081 | 0.605 | 0.211 | 0.697 | 0.632 | 0.161 | 0.287 | 0.371 | 0.844 | 0.840 |
| Visit 1 | 16.25(13.75,18.05) | 15.90(14.25,17.72) | 0.850 |  |  |  |  |  |  |  |  |  |  |  |  |  |  |
| Visit 2 | 15.65(14.48,18.55) | 16.20(14.6,17.80) | 0.978 |  |  |  |  |  |  |  |  |  |  |  |  |  |  |
| Visit 3 | 16.45(14.53,18.05) | 16.35(14.8,17.85) | 0.968 |  |  |  |  |  |  |  |  |  |  |  |  |  |  |
| Visit 4 | 15.70(14.60,17.65) | 16.80(14.87,17.37) | 0.703 |  |  |  |  |  |  |  |  |  |  |  |  |  |  |
| HCT |  |  |  | 0.319 | 0.456 | 0.268 | 0.605 | 0.126 | 0.140 | 0.938 | 0.537 | 0.747 | 0.240 | 0.225 | 0.360 | 0.444 | 0.627 |
| Visit 1 | 48.4(41.1,52.5) | 47.0(40.4,52.5) | 0.829 |  |  |  |  |  |  |  |  |  |  |  |  |  |  |
| Visit 2 | 46.8(42.8,52.4) | 47.3(41.6,52.2) | 0.757 |  |  |  |  |  |  |  |  |  |  |  |  |  |  |
| Visit 3 | 47.6(42.0,54.5) | 48.5(43.7,51.9) | 0.914 |  |  |  |  |  |  |  |  |  |  |  |  |  |  |
| Visit 4 | 47.3(43.0,52.1) | 49.6(43.9,51.2) | 0.796 |  |  |  |  |  |  |  |  |  |  |  |  |  |  |
|  |  |  |  |  |  | | | | | |  |  | | | | | |
|  |  |  |  |  |  | | | | | |  |  | | | | | |
| Variables/visits | Group 1 EAB-277  (n=21) | Group 2 Placebo  (n=20) | P  between group | P model  repeated measured | P different between visits within subjects of group 1 | | | | | | P model  repeated measured | P different between visits within subjects of group 2 | | | | | |
|  |  |  |  | group 1 | 1 vs 2 | 1 vs 3 | 1 vs 4 | 2 vs 3 | 2 vs 4 | 3 vs 4 | group 2 | 1 vs 2 | 1 vs 3 | 1 vs 4 | 2 vs 3 | 2 vs 4 | 3 vs 4 |
| MCV |  |  |  | 0.451 | 1.000 | 0.346 | 1.000 | 1.000 | 1.000 | 1.000 | 0.722 | 1.000 | 1.000 | 1.000 | 1.000 | 1.000 | 1.000 |
| Visit 1 | 70.90±4.48 | 72.16±3.54 | 0.331 |  |  |  |  |  |  |  |  |  |  |  |  |  |  |
| Visit 2 | 70.55±4.52 | 71.50±4.30 | 0.509 |  |  |  |  |  |  |  |  |  |  |  |  |  |  |
| Visit 3 | 70.46±4.37 | 72.09±3.60 | 0.206 |  |  |  |  |  |  |  |  |  |  |  |  |  |  |
| Visit 4 | 70.05±4.46 | 72.33±3.75 | 0.101 |  |  |  |  |  |  |  |  |  |  |  |  |  |  |
| MCH |  |  |  | 0.730 | 0.900 | 0.872 | 0.669 | 0.872 | 0.513 | 0.198 | 0.754 | 0.875 | 0.315 | 0.686 | 0.483 | 0.677 | 0.325 |
| Visit 1 | 24.15(23.05,25.25) | 24.85(23.82,25.52) | 0.304 |  |  |  |  |  |  |  |  |  |  |  |  |  |  |
| Visit 2 | 24.55(23.05,25.18) | 24.60(23.80,25.30) | 0.632 |  |  |  |  |  |  |  |  |  |  |  |  |  |  |
| Visit 3 | 24.55(22.73,24.90) | 24.70(23.60,25.35) | 0.457 |  |  |  |  |  |  |  |  |  |  |  |  |  |  |
| Visit 4 | 24.00(22.50,24.80) | 24.95(23.70,25.65) | 0.085 |  |  |  |  |  |  |  |  |  |  |  |  |  |  |
| MCHC |  |  |  | 0.157 | 0.670 | 0.445 | 0.712 | 0.638 | 0.570 | 0.010 | 0.737 | 0.896 | 0.872 | 0.952 | 0.739 | 0.943 | 0.631 |
| Visit 1 | 33.60(33.20,34.30) | 33.90(33.63,34.30) | 0.190 |  |  |  |  |  |  |  |  |  |  |  |  |  |  |
| Visit 2 | 33.60(33.20,34.60) | 33.90(33.70,34.20) | 0.165 |  |  |  |  |  |  |  |  |  |  |  |  |  |  |
| Visit 3 | 33.90(33.30,34.50) | 34.00(33.70,34.08) | 0.855 |  |  |  |  |  |  |  |  |  |  |  |  |  |  |
| Visit 4 | 33.50(33.30,33.88) | 34.00(33.70,34.30) | 0.031 |  |  |  |  |  |  |  |  |  |  |  |  |  |  |
| PLT |  |  |  | 0.938 | 0.601 | 0.809 | 0.910 | 0.872 | 0.959 | 0.438 | 0.852 | 0.432 | 0.086 | 0.167 | 0.888 | 0.687 | 1.000 |
| Visit 1 | 283(222,418) | 269(233,348) | 0.695 |  |  |  |  |  |  |  |  |  |  |  |  |  |  |
| Visit 2 | 309(185,440) | 280(237,380) | 0.800 |  |  |  |  |  |  |  |  |  |  |  |  |  |  |
| Visit 3 | 277(220,366) | 267(246,383) | 0.925 |  |  |  |  |  |  |  |  |  |  |  |  |  |  |
| Visit 4 | 294(238,406) | 274(246,379) | 0.737 |  |  |  |  |  |  |  |  |  |  |  |  |  |  |
| RDW |  |  |  | 0.809 | 0.678 | 0.827 | 0.975 | 0.887 | 0.629 | 0.605 | 0.321 | 0.032 | 0.155 | 0.225 | 0.548 | 0.760 | 0.759 |
| Visit 1 | 13.0(12.4,13.8) | 12.6(12.3,12.9) | 0.336 |  |  |  |  |  |  |  |  |  |  |  |  |  |  |
| Visit 2 | 12.8(12.5,13.6) | 12.9(12.5,13.4) | 0.966 |  |  |  |  |  |  |  |  |  |  |  |  |  |  |
| Visit 3 | 12.7(12.5,13.9) | 12.8(12.4,13.3) | 0.818 |  |  |  |  |  |  |  |  |  |  |  |  |  |  |
| Visit 4 | 13.1(12.3,14.0) | 12.8(12.2,13.5) | 0.419 |  |  |  |  |  |  |  |  |  |  |  |  |  |  |
|  |  |  |  |  |  |  |  |  |  |  |  |  |  |  |  |  |  |
|  |  |  |  |  |  |  |  |  |  |  |  |  |  |  |  |  |  |
|  |  |  |  |  |  |  |  |  |  |  |  |  |  |  |  |  |  |
| Variables/visits | Group 1 EAB-277  (n=21) | Group 2 Placebo  (n=20) | P  between group | P model  repeated measured | P different between visits within subjects of group 1 | | | | | | P model  repeated measured | P different between visits within subjects of group 2 | | | | | |
|  |  |  |  | group 1 | 1 vs 2 | 1 vs 3 | 1 vs 4 | 2 vs 3 | 2 vs 4 | 3 vs 4 | group 2 | 1 vs 2 | 1 vs 3 | 1 vs 4 | 2 vs 3 | 2 vs 4 | 3 vs 4 |
| ALB |  |  |  | 0.688 | 1.000 | 1.000 | 1.000 | 1.000 | 1.000 | 1.000 | 0.149 | 1.000 | 1.000 | 0.859 | 1.000 | 0.364 | 1.000 |
| Visit 1 | 3.04±0.40 | 3.12±0.31 | 0.501 |  |  |  |  |  |  |  |  |  |  |  |  |  |  |
| Visit 2 | 3.01±0.39 | 3.17±0.37 | 0.187 |  |  |  |  |  |  |  |  |  |  |  |  |  |  |
| Visit 3 | 3.06±0.46 | 3.11±0.33 | 0.718 |  |  |  |  |  |  |  |  |  |  |  |  |  |  |
| Visit 4 | 3.11±0.53 | 3.02±0.35 | 0.554 |  |  |  |  |  |  |  |  |  |  |  |  |  |  |
| ALT |  |  |  | 0.761 | 0.930 | 0.862 | 0.398 | 0.840 | 0.647 | 0.856 | 0.959 | 0.717 | 0.722 | 0.636 | 0.888 | 0.679 | 0.965 |
| Visit 1 | 44(33,67) | 47(36,79) | 0.663 |  |  |  |  |  |  |  |  |  |  |  |  |  |  |
| Visit 2 | 45(35,93) | 46(37,74) | 0.946 |  |  |  |  |  |  |  |  |  |  |  |  |  |  |
| Visit 3 | 46(35,73) | 47(40,88) | 0.633 |  |  |  |  |  |  |  |  |  |  |  |  |  |  |
| Visit 4 | 53(34,94) | 46(30,78) | 0.877 |  |  |  |  |  |  |  |  |  |  |  |  |  |  |
| ALP |  |  |  | 0.296 | 0.356 | 0.643 | 0.159 | 0.360 | 0.171 | 0.122 | 0.899 | 0.587 | 0.827 | 0.877 | 0.545 | 0.722 | 0.906 |
| Visit 1 | 49 (30,129) | 45(29,93) | 0.888 |  |  |  |  |  |  |  |  |  |  |  |  |  |  |
| Visit 2 | 44 (30,93) | 53(38,73) | 0.776 |  |  |  |  |  |  |  |  |  |  |  |  |  |  |
| Visit 3 | 51 (29,142) | 44(27,91) | 0.613 |  |  |  |  |  |  |  |  |  |  |  |  |  |  |
| Visit 4 | 40 (34,121) | 49(31,85) | 0.781 |  |  |  |  |  |  |  |  |  |  |  |  |  |  |
| BUN |  |  |  | 0.081 | 0.232 | 0.029 | 0.074 | 0.217 | 0.067 | 0.981 | 0.685 | 0.432 | 0.569 | 0.175 | 0.704 | 0.351 | 0.083 |
| Visit 1 | 23(18,29) | 17(13,20) | 0.034 |  |  |  |  |  |  |  |  |  |  |  |  |  |  |
| Visit 2 | 22(14,28) | 16(12,22) | 0.032 |  |  |  |  |  |  |  |  |  |  |  |  |  |  |
| Visit 3 | 18(13,26) | 15(12,20) | 0.341 |  |  |  |  |  |  |  |  |  |  |  |  |  |  |
| Visit 4 | 19(14,21) | 18(12,24) | 0.807 |  |  |  |  |  |  |  |  |  |  |  |  |  |  |
| CREA |  |  |  | 0.112 | 0.154 | 1.000 | 1.000 | 1.000 | 1.000 | 1.000 | 0.751 | 1.000 | 1.000 | 1.000 | 1.000 | 1.000 | 1.000 |
| Visit 1 | 1.02±0.16 | 0.94±0.31 | 0.639 |  |  |  |  |  |  |  |  |  |  |  |  |  |  |
| Visit 2 | 0.96±0.14 | 0.97±0.24 | 0.969 |  |  |  |  |  |  |  |  |  |  |  |  |  |  |
| Visit 3 | 1.01±0.29 | 0.97±0.18 | 0.100 |  |  |  |  |  |  |  |  |  |  |  |  |  |  |
| Visit 4 | 0.95±0.29 | 0.95±0.43 | 0.551 |  |  |  |  |  |  |  |  |  |  |  |  |  |  |
|  |  |  |  |  |  |  |  |  |  |  |  |  |  |  |  |  |  |
|  |  |  |  |  |  |  |  |  |  |  |  |  |  |  |  |  |  |
| Variables/visits | Group 1 EAB-277  (n=21) | Group 2 Placebo  (n=20) | P  between group | P model  repeated measured | P different between visits within subjects of group 1 | | | | | | P model  repeated measured | P different between visits within subjects of group 2 | | | | | |
|  |  |  |  | group 1 | 1 vs 2 | 1 vs 3 | 1 vs 4 | 2 vs 3 | 2 vs 4 | 3 vs 4 | group 2 | 1 vs 2 | 1 vs 3 | 1 vs 4 | 2 vs 3 | 2 vs 4 | 3 vs 4 |
| TP |  |  |  | 0.323 | 1.000 | 1.000 | 1.000 | 0.667 | 1.000 | 0.125 | 0.703 | 1.000 | 1.000 | 1.000 | 1.000 | 1.000 | 1.000 |
| Visit 1 | 6.8±0.8 | 6.7±0.6 | 0.592 |  |  |  |  |  |  |  |  |  |  |  |  |  |  |
| Visit 2 | 6.8±1.0 | 6.7±0.7 | 0.584 |  |  |  |  |  |  |  |  |  |  |  |  |  |  |
| Visit 3 | 7.0±1.0 | 6.7±0.7 | 0.288 |  |  |  |  |  |  |  |  |  |  |  |  |  |  |
| Visit 4 | 6.7±0.8 | 6.9±0.7 | 0.552 |  |  |  |  |  |  |  |  |  |  |  |  |  |  |
| GLO |  |  |  | 0.368 | 1.000 | 1.000 | 1.000 | .725 | 1.000 | 0.607 | 0.297 | 1.000 | 1.000 | 1.000 | 1.000 | 0.622 | 0.312 |
| Visit 1 | 3.8±0.8 | 3.6±0.7 | 0.423 |  |  |  |  |  |  |  |  |  |  |  |  |  |  |
| Visit 2 | 3.8±1.0 | 3.5±0.7 | 0.271 |  |  |  |  |  |  |  |  |  |  |  |  |  |  |
| Visit 3 | 3.9±1.1 | 3.6±0.8 | 0.268 |  |  |  |  |  |  |  |  |  |  |  |  |  |  |
| Visit 4 | 3.6±0.8 | 3.8±0.7 | 0.355 |  |  |  |  |  |  |  |  |  |  |  |  |  |  |
| A:G ratio |  |  |  | 0.467 | 1.000 | 1.000 | 0.718 | 1.000 | 1.000 | 1.000 | 0.051 | 1.000 | 1.000 | 0.138 | 1.000 | 0.124 | 0.335 |
| Visit 1 | 0.83±0.22 | 0.92±0.21 | 0.244 |  |  |  |  |  |  |  |  |  |  |  |  |  |  |
| Visit 2 | 0.85±0.24 | 0.95±0.25 | 0.200 |  |  |  |  |  |  |  |  |  |  |  |  |  |  |
| Visit 3 | 0.84±0.25 | 0.92±0.24 | 0.331 |  |  |  |  |  |  |  |  |  |  |  |  |  |  |
| Visit 4 | 0.91±0.27 | 0.82±0.17 | 0.283 |  |  |  |  |  |  |  |  |  |  |  |  |  |  |
| PP |  |  |  | 0.035 | 0.835 | 0.305 | 0.050 | 0.924 | 0.020 | 0.023 | 0.529 | 0.429 | 0.045 | 0.265 | 0.292 | 0.219 | 0.886 |
| Visit 1 | 8.3(8.0,9.0) | 8.1(8.0,8.9) | 0.649 |  |  |  |  |  |  |  |  |  |  |  |  |  |  |
| Visit 2 | 8.1(7.9,9.0) | 8.007.6,9.0) | 0.247 |  |  |  |  |  |  |  |  |  |  |  |  |  |  |
| Visit 3 | 8.0(8.0,8.8) | 8.0(7.8,8.2) | 0.186 |  |  |  |  |  |  |  |  |  |  |  |  |  |  |
| Visit 4 | 7.6(7.1,8.2) | 8.0(7.6,8,2) | 0.203 |  |  |  |  |  |  |  |  |  |  |  |  |  |  |

Reference

1	Häggström J, Boswood A, O’Grady M, Jöns O, Smith S, Swift S, et al. Effect of pimobendan or benazepril hydrochloride on survival times in dogs with congestive heart failure caused by naturally occurring myxomatous mitral valve disease: The QUEST study. J Vet Intern Med. 2008 Sep;22(5):1124–35.
