# Supplementary material for: Adjunctive Therapy With a Perna canaliculus and Euphausia superba Extract (EAB‐277TM) for Clinical Signs of Tracheal Collapse in Dogs: A Prospective, Randomized and Placebo‐Controlled Exploratory Trial
Source: Vet Med Sci. 2026 Aug 14;12(5):e71159. doi: 10.1002/vms3.71159 (PMC13475633; doi:10.1002/vms3.71159)
Supplement: Supplementary file 2 [file VMS3-12-e71159-s003.docx]

**Supplementary Figure legends**

**Supplementary Figure S1.** Representative fluoroscopic images illustrating changes in the tracheal lumen of a dog in the EAB-277™ treatment group between baseline and day 14. Images were obtained during inspiration and expiration: (A) day 0, inspiration; (B) day 0, expiration; (C) day 14, inspiration; and (D) day 14, expiration.
